# Social Cognition Deficits and Psychopathic Traits in Young People Seeking Mental Health Treatment

**DOI:** 10.1371/journal.pone.0067753

**Published:** 2013-07-04

**Authors:** Anita van Zwieten, Johanna Meyer, Daniel F. Hermens, Ian B. Hickie, David J. Hawes, Nicholas Glozier, Sharon L. Naismith, Elizabeth M. Scott, Rico S. C. Lee, Adam J. Guastella

**Affiliations:** 1 Brain & Mind Research Institute, University of Sydney, Sydney, NSW, Australia; 2 College of Medicine, University of Florida, Gainesville, Florida, United States of America; University of Granada, Spain

## Abstract

Antisocial behaviours and psychopathic traits place an individual at risk for criminality, mental illness, substance dependence, and psychosocial dysfunction. Social cognition deficits appear to be associated with psychopathic traits and are believed to contribute to interpersonal dysfunction. Most research investigating the relationship of these traits with social cognition has been conducted either in children or adult forensic settings. We investigated whether psychopathic traits were associated with social cognition in 91 young people presenting for mental healthcare (aged between 15 and 25 years). Participants completed symptom severity measures, neuropsychological tests, the Reading the Mind in the Eyes Test of social cognition (RMET), and the Antisocial Process Screening Device (APSD) to assess psychopathic personality traits. Correlation analyses showed poorer social cognition was associated with greater psychopathic traits (*r* = −.36, *p* = .01). Interestingly, social cognition performance predicted unique variance in concurrent psychopathic personality traits above gender, IQ sustained attention, and working memory performance. These findings suggest that social cognitive impairments are associated with psychopathic tendencies in young people presenting for community mental healthcare. Research is needed to establish the directionality of this relationship and to determine whether social cognition training is an effective treatment amongst young people with psychopathic tendencies.

## Introduction

Antisocial behaviors involve physical or psychological harm to others and include cheating, stealing, verbal or physical aggression, lying, overt or covert hostility, and other forms of criminality. A subgroup of antisocial adults also present with a constellation of psychopathic traits, which include affective (e.g., lack of empathy and guilt), interpersonal (e.g., use of others for self-gain), behavioral (e.g., impulsivity), and cognitive (e.g., inflated sense of self-importance) dimensions [1]. Antisocial behavior and psychopathic personality traits are associated with numerous comorbid mental health problems, including substance dependence [2], [3], [4], [5], problem gambling [6], suicidality [7], and anxiety disorders [5], [8], [9], [10]. They are also associated with social dysfunction in the form of interpersonal problems, unemployment, criminality, and aggression and violence towards others [5], [11].

Antisocial behaviors typically peak in late adolescence and early adulthood, with the expression of such behavior believed to increase ten-fold over this time [12]. According to Moffitt [12], [13], the development of life-course-persistent antisocial behaviour (as opposed to the more common short-term juvenile delinquency) reflects an ongoing interaction between the developmental environment and underlying abnormalities in neuropsychological, emotional, and personality factors. Individuals on this trajectory tend to engage in antisocial behaviours from childhood right through to later life, rather than simply in the form of juvenile delinquent behaviours during adolescence. Blair’s [14] neurocognitive Violence Inhibition Mechanism (VIM) model specifically describes how socio-cognitive skills and other neuropsychological factors may interact with environmental factors in the development of psychopathy, suggesting that the disorder reflects a disruption in the normal process of moral socialization. Normally, the pairing of others’ distress cues (the aversive unconditioned stimulus) with mental representations of the personal moral transgressions that caused the distress (the conditioned stimulus) acts to classically condition the individual against antisocial behaviour. Where this is learning is disrupted, there is insufficient development of a moral conscience which normally serves to inhibit antisocial behaviour, and promote empathic behaviour and moral emotions [1]. For instance, individuals who are insensitive to emotional expressions of fear and distress are unlikely to undergo the normal process of aversive conditioning to such cues during key periods of moral development.

Insensitivity to others’ emotional expressions (particularly negative emotions of distress and fear) and other social cognitive deficits have therefore been identified as a critical factor in the developmental pathway to psychopathy [14], [15], [16]. Social cognition encompasses a range of abilities such as the capacity to hold gaze and attend to relevant features of faces, recognize facial expressions of emotions [17], identify and attribute signals of social threat [18], and infer others’ mental state (i.e., Theory of Mind abilities) [19]. These social cognitive abilities are widely recognized as being crucial to socialization and normal social interaction [20], [21]. In the context of psychopathy, social cognitive deficits are relevant not only in terms of developmental pathways but also in potentially reducing the efficacy of behaviourally based treatment programs [22], [23].

A number of studies have examined the relationship between social cognition and psychopathic traits in child and adolescent samples. One widely-used measure of psychopathic traits is the Antisocial Process Screening Device or APSD [24], which assesses callous/unemotional personality traits, narcissism, and impulsivity. In previous studies amongst children and adolescents, APSD scores have been found to be associated with deficits in emotional stimulus processing [25], [26], as well as outcome expectancies and values in relation to aggressive behaviour [23]. One study by Blair and colleagues [15] showed that boys with greater psychopathic tendencies, as indicated by higher APSD scores, had a selective impairment for recognition of fearful vocal affect. Meanwhile, scores on the Callous-Unemotional subscale of the APSD have been shown to be associated with deficits in Theory of Mind [27], emotional processing and recognition [28], [29], [30], [31], [32], social problem solving skills [33], and the capacity for social eye gaze [30].

A limitation of existing research in this field is the lack of studies conducted in older adolescent and young adult populations. This is particularly surprising given the peak in anti-social behaviour across late adolescence and into young adulthood [12], increasing knowledge of the importance of successful transitions during this particular development period for longer term health, and the recent developments of targeted mental health services for this age group [34], [35].

In spite of the strong association between antisocial behaviour, psychopathic tendencies, and mental health problems, the relationship between APSD scores and social cognition has not been examined in young mental health treatment-seeking samples. Previous studies have been conducted in samples of children recruited from the community, juvenile offenders or children referred specifically for conduct or behavioral problems. The few studies that have examined relationships between social cognition and antisocial behaviours or psychopathy in adults have used incarcerated offenders [36], [37], [38], [39]. This makes it difficult to draw conclusions about the value of research outcomes to community mental health settings where the majority of clinicians practice.

The purpose of this study was to assess the relationship between psychopathic personality traits and social cognition in a group of young people seeking treatment from a youth mental health service. Given the association between psychopathic traits and neuropsychological deficits [40], we included a number of neuropsychological tests as control variables to ensure that differences in social cognition performance across levels of psychopathic traits would not be confounded with differences in these skills. We also included demographic variables of gender and IQ which have known relationships with psychopathic traits [41], [42]. Based on previous research, it was hypothesized that social cognitive performance would predict the concurrent presence of psychopathic traits over and above demographic and neuropsychological variables.

## Materials and Methods

### Ethics Statement

The current study was approved by the University of Sydney Human Research Ethics Committee. After a complete description of the study to the participants, including a standard Participant Information Statement, those aged 16 years and above gave written informed consent to participate, whereas for those under the age of 16 years both the participant and their legal guardian gave written informed consent in accordance with Human Research Ethics Committee guidelines and Australian law. Consent was undertaken using a standard Participant or Guardian Consent Form. All participants were informed during the consent process that their decision whether to participate would not affect their treatment or relationship with the clinician or researchers, and of their right to withdraw without penalty. All informed consent paperwork was approved by the University of Sydney Human Research Ethics Committee.

### Participants

Recruitment occurred at *headspace*, a specialized tertiary community referral service for the assessment and early intervention of young people with mental health problems in the inner west of Sydney [34], [35]. Participants included 91 young persons between 15 and 25 years of age presenting for mental healthcare, who were selected on the basis of willingness to participate in more detailed neuropsychological, neuroimaging and longitudinal follow-up studies. Exclusion criteria were medical instability (as determined by a psychiatrist), history of neurological disease (e.g., tumor, head injury, epilepsy), medical illness known to impact cognitive and brain function (e.g., sleep apnea), electroconvulsive therapy (ECT) in last 3 months, intellectual and/or developmental disability (a predicted IQ score <70), primary diagnosis of a psychotic illness, or insufficient English language skills. 54% of participants presented with a primary diagnosis of a depressive illness, 32% with a primary diagnosis of bipolar disorder I or II, 9% with an anxiety disorder (including obsessive compulsive, generalized anxiety, agoraphobia/panic, and social anxiety disorders), and 5% with a primary diagnosis outside these categories (including personality disorders, behavioral difficulties, and attention deficit hyperactivity disorder).

### Procedure

Participants were given all forms and self-report questionnaires to complete in the waiting room and then completed a structured clinical interview to confirm DSM-IV diagnosis [43], a social cognitive assessment, and a neuropsychological assessment on the same day. Questionnaires and neuropsychological assessment were part of a larger battery of tests within a program of longitudinal youth mental health research [44]. As described elsewhere [44], [45], the clinical assessment was undertaken by clinical psychiatrists, clinical psychologists, mental health nurses, or medical practitioners with training in mental health.

### Measures: Self-Report

#### Antisocial Process Screening Device (APSD)

The Antisocial Process Screening Device (APSD) [24] is a 20-item self-report measure of psychopathic traits in youth, adapted from the original parent/teacher-report APSD developed for preadolescent child samples. Research shows that from childhood to adolescence, the validity of self-report measures of psychopathology increases and that psychopathy remains stable from early adolescence into young adulthood [46]. Each item is rated on a 3-point Likert-type scale as 0 *(not at all true)*, 1 *(sometimes true)*, or 2 *(definitely true)*, with four items reverse scored. Confirmatory factor analysis has shown the self-report version of the APSD to consist of 3 subscales: Narcissism (items 5, 8, 10, 11, 14, 16), Impulsivity (items 1, 4, 9, 13, 17), and Callous-Unemotional (CU) traits (items 3, 7, 12, 18–20), with two items (items 2 and 6) not loading on any factor [47], [48]. This 18 item three-factor structure was used in the analyses for the present study. By summing scores across the subscales (excluding items 2 and 6), a total score is generated. The construct validity of the APSD has been established in community- and clinic-based samples [49], [50], [51], as well as delinquent populations [47]. High APSD scores have been shown to be associated with early onset offending [52], number of arrests and violent arrests [5], greater variety and frequency of violence [53], [54], lack of sensitivity to punishment in social situations [23], aggression and administrative infractions, as well as poor treatment outcome in adjudicated samples [55]. Research with forensic populations has shown that total scores on the youth version of the Psychopathy Checklist or PCL-YV [56] correlate significantly and positively with total scores on the youth-report form of the APSD, however these correlations have been only modest in numerous samples [47], [51]. The APSD shows strong test-retest reliability and internal consistency [50], [57], [58]. In this sample, the internal consistency for the APSD total score was α = 0.82.

#### Depression Anxiety Stress Scale (DASS)

The Depression Anxiety Stress Scale-21 [59] is a 21 item self-report measure that assesses depression, anxiety, and tension/stress over the past week. Each of the three subscales (Depression, Anxiety, and Stress) includes 7 Likert-type items rated on a 4-point scale between 0 (*Did not apply to me at all*) and 3 (*Applied to me very much, or most of the time*). Higher total scores indicate more frequent occurrence of the relevant symptoms. The DASS is a psychometrically sound instrument with good internal consistency in both clinical and nonclinical samples [59], [60]. The factor structure of the DASS-21 is stable and it has good convergent and discriminant validity [61], [62], [63].

#### Alcohol Use Disorders Identification Test (AUDIT)

The Alcohol Use Disorders Identification Test [64], [65] was created by the World Health Organization to identify hazardous or harmful drinking habits. The AUDIT consists of ten self-report items that assess three domains of alcohol consumption (items 1–3), dependence (items 4–6) and adverse consequences related to alcohol use (items 7–10). Items are rated from 0 to 4, in terms of frequency of occurrence, to yield a total score ranging between 0 and 40. Scores of 8 or more are taken as an indicator of ‘risky drinking’ with a moderate risk of harm, 16–19 a higher-risk or ‘harmful’ level of drinking, and scores of 20–40 indicate ‘high-risk’ drinking. It has strong internal consistency and test-retest reliability [66], [67], [68].

### Measures: Social Cognitive and Neuropsychological Assessment

#### Reading the Mind in the Eyes Test (RMET)

The Reading the Mind in the Eyes Test [69] is a measure of social cognitive capacity that assesses the ability to attribute mental states to others by looking at photographs of eye regions of human faces. Participants were asked to observe 36 images, and decide which of four mental states best described what each person is feeling or thinking. Definitions of each word were provided to reduce potential confounding with vocabulary skills. A total score was produced by adding up the number of correct answers and converted to a percentage correct score. A higher score indicates a greater capacity to identify others’ mental states (i.e., Theory of Mind). The RMET has been used successfully in both nonclinical and clinical samples [69], [70], [71], [72]. It is sensitive to individual differences in social cognitive abilities and is not subject to ceiling effects amongst healthy populations as is common with other measurements of social cognition [69].

#### Intellectual Function (IQ)

Predicted IQ was assessed using either the Wechsler Test of Adult Reading (WTAR) [73] for participants aged 16–25 years, or the Wide Range Achievement Test 4 (WRAT-4) [74] for participants below 16 years of age.

#### Trail making test

The Trail Making Test (TMT) assesses psychomotor speed and set-shifting ability [75]. Part A (TMT-A) assesses visual scanning, number recognition, numeric sequencing, and motor speed. Part B (TMT-B) also assesses set shifting by having the participant draw connecting lines alternating between consecutive numbers and letters (e.g., 1, A, 2, B, 3, C). The amount of time required to complete each task is considered the individual’s raw score. The TMT has been used for neurological assessment in a variety of populations [76], [77], [78], [79].

#### Rapid Visual Information Processing Task (RVP)

The Rapid Visual Information Processing task (RVP) from the Cambridge Neuropsychological Test Automated Battery (CANTAB) [80] was employed as a measure of sustained attention. Participants were presented with random digits on a computer monitor, at a rate of 100 digits per minute. A response bar was pressed as quickly as possible when the participant identified a string of three consecutive odd or even numbers. Eight target triplets occur every minute during the four-minute task. The RVP-A score relates to omission errors (misses), whereas the RVP-B relates to commission errors (false positives). This task has been shown to be a valid assessment of sustained attention in both healthy individuals and those with neuropsychiatric disorders [80].

#### Spatial Span Test (SSP)

The Spatial Span Test (SSP) from the CANTAB [80] was employed as a measure of working memory capacity. In this test the participant watched white squares change color and touched the boxes that changed color in the same order in which they were displayed. The outcome measure of interest was spatial span length (the longest forward sequence recalled successfully).

### Data Analysis

Statistical analyses were performed using SPSS for Windows 20.0. Missing values were replaced with the series mean. If homogeneity of variance was violated (according to Levene’s test) the corrected degrees of freedom and p-values were reported using Welch’s procedure. Neuropsychological variables were converted to ‘demographically corrected’ standardized scores (i.e. z-scores) using established norms. Prior to analyses, outliers beyond ±3.0 z-scores for each neuropsychological variable were curtailed to values of +3.0 or −3.0 (depending on the direction) so that individuals with extreme scores did not influence results of between-group tests. There were no outliers beyond −/+3.0 for RVP-A, SSP, and RVP-B. The number of cases beyond −3.0 for TMT A and TMT-B did not exceed 5%. As the distribution of the TMT-B variable was highly skewed, even after curtailing, it was excluded from analyses.

## Results

### Psychopathic Traits, Social Cognition and Mental Health Variables

Participants were 62 females and 29 males aged between 15 and 25 with a mean of 19.98 (*SD = *2.74) years. Demographic characteristics and predicted IQ for the sample are presented in Table 1. In order to explore relationships between demographic characteristics, Pearson correlations of both RMET and APSD scores were run with age, predicted IQ, and symptom severity measures and are presented in Table 2. Higher scores on the APSD were associated with lower predicted IQ and increased AUDIT total scores. In contrast, APSD scores were not significantly associated with any of the DASS subscales. RMET scores exhibited a significant positive correlation with predicted IQ. Age showed no significant association with either RMET or APSD score. Independent samples t-tests demonstrated that females and males did not differ significantly on APSD scores (*t* (89)_ = _1.6, *p = *.113) or on RMET scores (*t* (44)* = *−2.00, *p = *.052). For RMET scores, Levene’s test indicated unequal variances, *F* (1, 89)* = *5.06, *p* = .027, so degrees of freedom were adjusted from 89 to 44. These results are presented in Table 3.

**Table 1:** Demographic Characteristics and IQ of Total Sample and Quartile Groups of APSD Total Score.

APSD Score Quartile	Gender	Age	Predicted IQ
*M (SD)* APSD Score	% Female (*n)*	*M (SD)*	*M (SD)*
**1^st^** a			
*M = *5.19 (1.46)	70.8 (17)	19.84 (2.61)	106.42 (6.63)
**2^nd^** b			
*M = *9.49 (1.32)	79.2 (19)	20.01 (2.68)	105.71 (8.62)
**3^rd^** c			
*M = *14.02 (1.45)	63.6 (14)	20.57 (2.95)	102.60 (7.80)
**4^th^** d			
*M = *20.05 (2.77)	57.1 (12)	19.49 (2.82)	103.25 (8.52)
**Total Sample**			
*M = *11.89 (5.77)	68.1 (62)	19.98 (2.74)	104.58 (7.98)

Note.

a n = 24.

b n = 24.

c n = 22.

d n = 21.

**Table 2:** Correlations of RMET and APSD with Demographic, Neuropsychological and Symptom Severity Measures.

	AUDIT	DASS Stress	DASSAnxiety	DASSDepression	Age	IQ	TMT A	RVP A	RVP B	SSP
APSD	.377**	.136	.105	.133	−.014	−.229*	−.044	−.266*	−.183	−.321**
RMET	−.119	−.169	−.132	−.149	−.096	.426**	.276**	.265*	.238*	.208*

*Note. n = *91. All correlations are two-tailed.

* *p<*0.05.

** *p<*0.01.

**Table 3:** APSD and RMET Total Scores by Gender.

	Male^a^	Female^b^
RMET % Correct	0.72 (0.14)	0.78 (0.10)
APSD Total Score	13.29 (5.86)	11.23 (5.66)

Note.

a n = 29.

b n = 62.

The APSD was also split into quartile scores and demographic and symptom severity scores were evaluated within each quartile (See Tables 1 and 4). Univariate ANOVAs confirmed that there were no significant differences across quartile groups in predicted IQ, *F* (3, 87) = 1.24, *p = *.300, or age, *F* (3, 87) = 0.58, *p = *.632. Similarly, chi-square analysis revealed no significant differences in the distribution of gender across quartile groups of APSD scores, *χ^2^* (3, 91)* = *2.80, *p = *.424. Differences in symptom severity scores across quartiles of APSD total score were then analyzed using univariate ANOVAs (see Table 4). Consistent with the correlations presented above, there were no significant differences in DASS subscale scores across quartiles of APSD score. However, there were significant differences in AUDIT total scores across quartile groups, *F* (3, 87) = 4.25, *p = *.008. Polynomial contrast analysis revealed a significant linear trend for AUDIT scores across APSD quartiles, *F* (1, 87) = 11.63, *p = *.001, whilst quadratic and cubic trends were not significant. As can be seen through examination of mean AUDIT scores across quartiles, AUDIT scores increased across increasing quartiles of APSD score.

**Table 4:** Symptom Severity Scores Across Quartiles of APSD Score.

APSD Score Quartile	DASS Stress	DASS Anxiety	DASS Depression	AUDIT Total
1^st^ a	18.38	12.23	18.31	5.83
	(9.36)	(9.59)	(8.53)	(5.74)
2^nd^ b	23.60	16.96	23.73	6.49
	(8.10)	(8.76)	(12.43)	(5.96)
3^rd^ c	21.16	15.93	17.60	8.26
	(6.64)	(7.40)	(9.89)	(6.37)
4^th^ d	22.29	14.95	24.76	12.03
	(12.04)	(10.71)	(12.88)	(7.30)
All e	21.33	14.99	21.05	8.02
	(9.24)	(9.19)	(11.31)	(6.67)
	*F* (3, 87) = 1.39	*F* (3, 87) = 1.17	*F* (3, 87) = 2.47	*F* (3, 87) = 4.25
	*p>*0.05	*p*>0.05	*p*>0.05	*p = *.008

Note.

a n = 24.

b n = 24.

c n = 22.

d n = 21.

e n = 91.

### Psychopathic Traits, Social Cognition and Neuropsychological Performance

Pearson’s correlations between the neuropsychological test variables, social cognition, and APSD scores are presented in Table 2. Only the RVP-A and SSP were significantly associated (both in a negative direction) with psychopathic traits as measured using the APSD (all other *p* values >0.05). All neuropsychological test scores were significantly associated with social cognition performance on the RMET.

**Figure 1:**
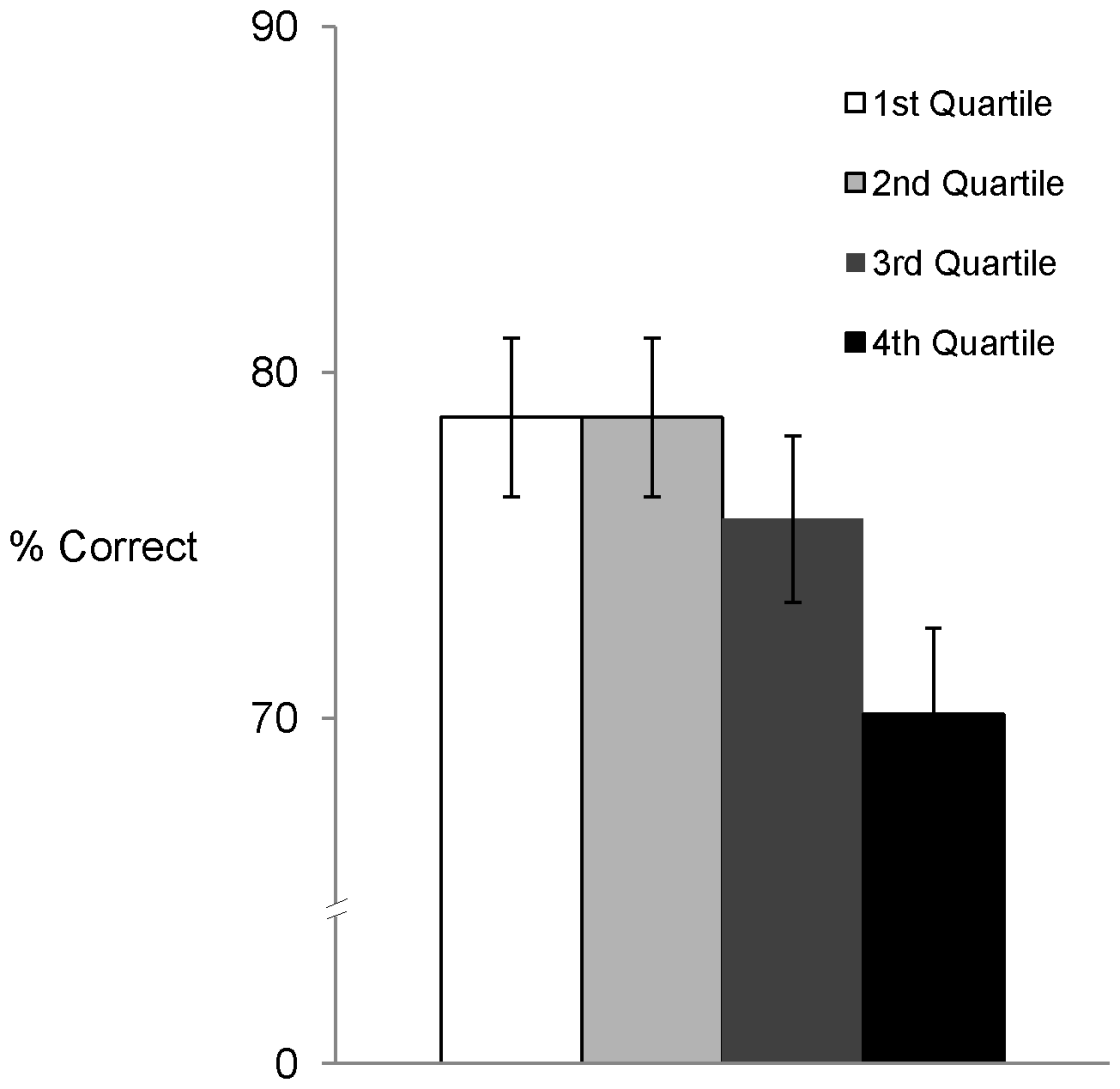
Mean Percentage of Items Correct on RMET Across Quartile Groups of APSD Total Score. *Note. Data presented are mean ± standard errors of the mean (SEM).*

Results showed a significant negative association between APSD and RMET scores, *r* (91) = −0.36, *p = *0.001, such that higher levels of psychopathic traits were associated with poorer performance on the RMET social cognition test. Univariate ANOVA was used to perform a preliminary investigation into the relationship between RMET performance and psychopathic traits as represented in quartiles of APSD total score. This revealed significant differences in RMET performance across APSD quartiles, *F* (3, 87) = 2.78, *p = *.046, and a significant linear trend across quartiles, *F* (1, 87) = 7.11, *p = *.009. As can be seen in Figure 1, this reflected a decrease in the mean percentage of correct items on the social cognition test across increasing quartiles of APSD score.

Based on the above set of relationships, a forced-entry hierarchical linear regression was run to assess the capacity of performance on the social cognition test (percentage correct on the RMET) to predict psychopathic traits (APSD total score), when controlling for gender, predicted IQ, and performance on the SSP and RVP-A. RVP-A and SSP were included as they were the only neuropsychological test variables to exhibit a significant correlation with APSD score. As mentioned above, TMT-B was excluded at the outset of data analysis because of its skewed distribution. Age was not included as a control variable because it did not show a significant correlation with either APSD or RMET score. Gender and predicted IQ were entered in the first step of the regression and together accounted for a significant 7.9% of variance in APSD score, *F* (2, 88) = 3.77, *p = *.027. The addition of RVP-A and SSP score in the second step resulted in a significant increase in the proportion of variance in APSD score explained by the model, Δ*F* (2, 87) = 4.73, *p = *.011, Δ*R* ^2^ = 0.091. The addition of RMET score in the final step resulted in a significant increase in the proportion of variance in APSD score explained by the model, Δ*F* (1, 85) = 4.38, *p = *.039, Δ*R* ^2^ = 0.041. That is, RMET scores explained an additional 4.1% of variance in psychopathic traits above that which was accounted for by control variables alone. The overall model predicting APSD score from gender, predicted IQ, RVP-A score, SSP score, and RMET score was significant, *F* (5, 85) = 4.55, *p = *.001. Altogether 21.1% of variance in APSD scores was accounted for, and model characteristics are presented in Table 5. When the regression was run with the other neuropsychological test scores that did not exhibit significant correlations with APSD score (TMT-A and RVP-B) in the second step, RMET performance still accounted for a significant proportion of variance in APSD score beyond the control variables (Δ*F* (1, 83) = 4.63, *p = *.034).

**Table 5:** Hierarchical Regression Predicting APSD Score from Gender, IQ, RVP-A, SSP, and RMET Score.

Predictor	Predictor B	Model ΔR^2^
Model 1: Demographics		
Gender	−2.01	
IQ	−0.16*	
		0.08*
Model *F* (2, 88) = 3.77, *p = *.027		
Model 2: Neuropsychological		
Gender	−1.79	
IQ	−0.11	
RVP-A score	−0.88	
SSP score	−1.20*	
		0.09*
Model *F* (4, 86) = 4.41, *p = *0.003		
Model 3: Social Cognition		
Gender	−1.14	
IQ	−0.04	
RVP-A	−0.69	
SSP	−1.11	
RMET	−11.53*	
		0.04*
Model *F* (5, 85) = 4.55, *p = *0.001		Total R^2^ = 0.21

*Note.* *p<0.05.

Given that APSD total scores index psychopathic traits in terms of CU traits in combination with general conduct problems, post-hoc analyses were then conducted to examine the respective subscales of the APSD that map onto Hare’s [81] two-factor model of psychopathy (CU traits; impulsive conduct problems). This allowed us to determine whether the significant effect seen for APSD total scores was primarily accounted for by either CU traits or general conduct problems. Significant inverse correlations were found between RMET scores, and both CU traits (*r* = −.28, *p* = <.01) and impulsive conduct problems (*r* = −.32, *p*<.01). The comparable size of these correlations suggest that levels of CU traits as well as general conduct problems both contributed significantly to the effect seen for APSD total scores in the substantive analysis. As such, it does not appear that the poorer Theory of Mind abilities of participants with higher APSD total scores were simply an artefact of increased conduct problem severity among those individuals.

## Discussion

The current study assessed the relationship between psychopathic traits and social cognition performance in a sample of young adults seeking mental health treatment. This study firstly shows that psychopathic traits were associated with an increase in risky drinking as assessed by the AUDIT. Second, the results show a potentially important relationship between psychopathic traits and social cognition performance in a youth community mental health sample, such that participants who scored more highly on the APSD showed poorer social cognitive performance. When placed into a predictive model, social cognition performance accounted for a unique proportion of variance in psychopathic traits, even when controlling for working memory and sustained attention performance, IQ and gender.

Previous studies have shown that children high in psychopathic traits show deficits in various aspects of social cognition [25], [26], [27], [28], [29], [30], [31], [32], [33]. The present findings extend this relationship to a sample of mental health treatment-seeking young people in an older age bracket of 15 to 25. We note that while the majority of previous studies have highlighted deficits in fear recognition or social problem solving, this study suggests specific deficits in Theory of Mind, as assessed by the RMET. In this sense, it corroborates Stellwagen and Kerig’s [27] findings with a verbal Theory of Mind test in children. As our design was cross-sectional, we cannot draw conclusions about the causal nature of the relationship between social cognition and psychopathic traits as operationalized in the APSD. This is complicated further by the fact that there are theoretical grounds to believe that this relationship is of a complex and bidirectional nature. Just as social cognitive deficits may interfere with socialization and so contribute to the development of antisocial behaviours and psychopathic traits; so those with psychopathic tendencies are less likely to develop social cognitive skills or be motivated to employ those skills to decipher the internal states of others [27]. Further studies employing longitudinal designs are needed to explore this relationship.

As noted by Shamay-Tsoory et al. [82], the question of whether psychopathic traits are associated with deficits in Theory of Mind abilities has been the subject of some controversy. In contrast to the association between psychopathic traits and performance on the RMET found in the current study, at least two previous studies have failed to identify such an association [36], [39]. Numerous factors may potentially account for differences in the findings of these studies. First, whilst the sample in the current study was recruited from referrals to an outpatient psychiatric clinic, the studies by Dolan and Fullam [36] and Richell et al. [39] were both conducted with incarcerated offenders. Second, while the age range of our sample was largely adolescent, the samples examined in these two previous studies extended significantly into adulthood. Third, research across a range of domains related to psychopathy has shown that different measures of psychopathic traits demonstrate somewhat distinct associations with correlates of psychopathy [83]. As these previous adult studies both used the PCL-R, it is important that future research examines this association in both clinical and forensic samples of youth, using a range of established psychopathy measures. Interestingly, while Dolan and Fullam [36] did not find an association between psychopathic traits and RMET scores, the study did find that both psychopathic and non-psychopathic offenders performed worse on subtle tests of mentalizing ability (i.e., faux pas tasks) than healthy controls.

The current results highlight the potential to modify treatment programs for those who display psychopathic traits in youth clinical mental health services. The current research suggests that treatment regimens aimed at increasing social functioning in young persons with psychopathic traits could also focus on improving social cognitive abilities such as emotion recognition, social problem solving, and eye contact [29], [84]. Previous studies have shown that in order to maximize treatment gains, children high in psychopathic traits and antisocial behaviours require specialized or enriched treatment that involves implementing a token economy [85], raising the intensity of treatment [86], and focusing on rewarding experiences within the context of a positive parent-child relationship [87]. Future research in this domain should utilize longitudinal treatment outcome designs in order to assess the capacity of social cognitive, positive and reward-based treatment regimens to improve social functioning in young adults with psychopathic traits.

This study adds to existing literature by providing support for the utility and construct validity of the APSD as a screening device for psychopathic traits in youth mental health samples in addition to samples of incarcerated youth or juvenile offenders. Not only did correlational analyses show that youth in this sample who exhibited more psychopathic tendencies reported more alcohol-related problems, but in comparison with the first two quartiles of APSD score, those in the 3^rd^ and 4^th^ quartiles obtained AUDIT scores above the accepted cutoff for risky drinking. This finding is consistent with the literature surrounding associations between substance misuse and psychopathic traits [88] and with high rates of substance misuse reported in youth mental health treatment-seeking populations more broadly [89]. This further underscores the need to consider substance misuse in treatment approaches amongst this population.

Aside from its cross-sectional nature, a number of limitations of the study must be acknowledged. Only one facet of social cognition, Theory of Mind, was assessed in this study because of time constraints. Although the RMET is a psychometrically sound measure of emotion recognition, it does not tap into other areas of social cognition, such as gaze perception, prosody, and sarcasm identification. Similarly, the neuropsychological assessment included assessments of IQ, attention, and working memory. However, other aspects of neuropsychological performance which may have important relationships with social cognition and psychopathic traits (such as abstract reasoning, judgment, impulsivity, comprehension, and insight) were not included. Further, the APSD is a measure of psychopathic traits rather than specific antisocial behaviours. Although the APSD has been shown to correlate well with these outcomes, future studies should include measures such as externalizing behaviour and criminality to examine their relationships with social cognition alongside psychopathic traits as measured by the APSD. Finally, power for the correlational and regression analyses was likely limited by the relatively small sample size.

In summary, this study extended the relationship between psychopathic traits and social cognition deficits that has been found in children [15], [23], [27], [29], [90], [91] to an older sample of young people presenting for mental healthcare aged 15 to 25 years. It also highlighted alcohol misuse amongst individuals presenting with higher levels of psychopathic traits. Just as children high in psychopathic traits require specialized treatment [85], [86], [87], these results suggest that young persons with psychopathic traits in general mental health settings may present with unique social cognitive deficits that are relevant to treatment practice and the development of interventions.
